# Duchenne Muscular Dystrophy Under Three Years of Age

**DOI:** 10.3390/children13070857

**Published:** 2026-06-27

**Authors:** Ayşe Nur Coşkun, Haluk Topaloğlu

**Affiliations:** 1 Pediatrics Clinic, Ankara Atatürk Sanatoryum Training and Research Hospital, Ankara 06290, Türkiye; 2 Department of Pediatrics, Faculty of Medicine, Yeditepe University, İstanbul 34755, Türkiye

**Keywords:** creatine kinase, developmental delay, Duchenne muscular dystrophy

## Abstract

**Highlights:**

**What are the main findings?**

- Over 90% of DMD patients under three years of age are diagnosed incidentally through hyperCKemia, while nearly one-third present with early language development delays.
- Definitive confirmation was secured in 100% of cases, with 99% verified via molecular genetics and 1% via dystrophin-deficient muscle biopsy.

**What are the implications of the main findings?**

- In countries without newborn screening, routine screening of creatine kinase (CK) levels in infants with non-motor delays provides a critical window for early diagnosis.
- Neurodevelopmental surveillance must be standardized as an integral, core component of early childhood DMD management alongside motor monitoring.

**Abstract:**

**Background/Objectives**: Duchenne muscular dystrophy (DMD) is a progressive X-linked neuromuscular disorder. This retrospective study evaluated the demographic, genetic, and clinical characteristics of children diagnosed with DMD before age three to understand early clinical presentation profiles. **Methods**: The cohort included 198 boys diagnosed with DMD before three years of age between January 2020 and July 2025. Medical records, serum creatine kinase (CK) levels, language milestones via Denver II criteria, and multi-exon deletion maps were retrospectively evaluated. **Results**: Regarding the diagnostic entry pathways, the initial clinical trigger that led to medical investigation was incidental hyperCKemia in 91.4% of cases. Regardless of the presentation trigger, a definitive, confirmed diagnosis was established in all 198 cases: 196 patients (99.0%) were securely confirmed via genetic testing (MLPA or sequencing), while 2 patients (1.0%) with negative genetic panels were confirmed via muscle biopsy demonstrating a complete absence of dystrophin expression. Genetic analysis revealed deletions in 77.8% of patients, predominantly multi-exon deletions clustered in the distal hotspot region. Independent ambulation occurred at a median age of 16 months, and 14.6% achieved walking after 18 months. Delayed language development was observed in 29.8% of patients. **Conclusions:** Our findings indicate that early childhood DMD is characterized not only by early muscle involvement but also by prominent neurodevelopmental features. These findings underscore the value of early CK screening in young boys and support integrating standardized neurodevelopmental surveillance into early DMD care.

## 1. Introduction

Duchenne muscular dystrophy (DMD) is a progressive neuromuscular disorder that primarily affects boys and is caused by mutations in the dystrophin gene [1]. The dystrophin gene is a large gene on the X chromosome that is crucial for the production of dystrophin [2]. Dystrophin is an essential structural protein in muscle tissue, playing a key role in maintaining muscle integrity. A deficiency of dystrophin causes a gradual loss of skeletal muscle, leading to decreased motor function and, ultimately, premature death [3]. In addition to its role in muscle, dystrophin is expressed in the brain, and its deficiency may result in early neurodevelopmental manifestations such as delayed walking, speech delay, or autism spectrum features [4,5].

Early diagnosis and intervention are crucial in managing DMD, as they enable the timely initiation of multidisciplinary care, facilitate early access to emerging disease-modifying therapies, and allow for anticipatory guidance and family counseling [6,7]. Early identification also provides a critical window for monitoring neurodevelopmental trajectories and implementing supportive interventions before irreversible functional decline occurs [8]. Elevated creatine kinase (CK) levels often represent the earliest biochemical indicator of the disease, and the widespread availability of rapid, highly sensitive genetic testing now allows for a definitive molecular diagnosis within a relatively short time frame [9,10,11]. In parallel, increasing global awareness has improved the recognition of early clinical and biochemical signs of DMD [12]. Nevertheless, despite these advances, many children continue to be diagnosed later than optimal, typically after the age of four [10,13]. This study aims to investigate the demographic characteristics, as well as the genetic and clinical features of DMD patients diagnosed before three years of age.

## 2. Materials and Methods

This retrospective study was conducted between January 2020 and July 2025 at the Pediatric Neurology Outpatient Clinic of Yeditepe University. Consequently, the cohort comprised 198 pediatric patients diagnosed with DMD under three years of age. Patients whose DMD diagnosis was verified by genetic analysis or muscle biopsy were included in the study. The data of the patients included in this study were extracted retrospectively from medical records. No additional diagnostic procedures, clinical assessments, or physical interventions were performed, and all patient identifiable data were completely anonymized. While standard institutional guidelines did not originally mandate formal board review for non-invasive retrospective chart reviews, a formal application has been submitted to the institutional review board to comply with international publishing standards.

In accordance with national healthcare documentation protocols and institutional privacy regulations governed by the Personal Data Protection Law in Türkiye, descriptive metrics regarding race and specific ethnicity are not collected or categorized within electronic medical databases. Hence, the cohort represents a homogeneous regional sample of Turkish nationals. The patients’ data were collected retrospectively from medical records, including age, month of diagnosis, diagnostic methods, serum creatine kinase (CK) levels, presence of calf muscle hypertrophy at diagnosis, maternal carrier status, family history, genetic test results, age at walking, and language development milestones. Calf muscle pseudohypertrophy was noted by the pediatric neurologist during clinical observation. To ensure consistency and minimize inter-observer bias, all physical examinations and developmental documentations were directly supervised by the same senior pediatric neurologist, who specializes in neuromuscular disorders. Given the retrospective nature of the study, initial serum CK measurements were not exclusively performed at our institution, with some values originating from external referring centers. In this cohort, incidental CK elevation was defined as hyperCKemia initially discovered during routine laboratory investigations ordered for non-specific pediatric reasons, including standard well-child surveillance, preoperative screening, or acute febrile illnesses. Because the study retrospectively enrolled patients with a molecularly confirmed diagnosis of DMD, patients presenting with transient hyperCKemia of alternate etiologies were not tracked.

The genetic results of these patients were retrospectively evaluated. No additional genetic testing was performed specifically for this study. It was observed that, during the diagnostic process, multiplex ligation-dependent probe amplification (MLPA) was performed to assess deletions and duplications. Based on the MLPA results, some patients subsequently underwent comprehensive DMD gene sequencing and/or whole-exome sequencing (WES). According to the MLPA results, the deletions in the affected boys were categorized by their structure as single-exon deletions (affecting one exon), multi-exon deletions (affecting 2–10 exons), and large deletions (involving more than 10 exons) [14,15]. In addition, according to the literature, deletions were further classified based on DMD gene hot-spot regions as follows: the proximal hot-spot region (5′ region), involving exons 2–20; the distal (central) hot-spot region (3′ region), involving exons 45–53; non-hot-spot regions, involving exons 1–2 and 54–79; and the intermediate region, involving exons 21–44 [11,15]. In patients with point mutations, mutation types were classified into three categories: nonsense, frameshift, and splice-site mutations.

Maternal carrier screening was systematically performed for the mothers of all enrolled patients (*n* = 198 mothers evaluated in total) as part of subsequent family counseling protocols. In accordance with standard genetic counseling protocols, maternal carrier testing was performed retrospectively after the diagnosis was firmly established in the index patient, with the exception of one case where the mother’s carrier status was known prior to the index pregnancy. Family history was documented based on a known pedigree of DMD in maternal relatives.

Since the study was conducted retrospectively, a standardized developmental screening test could not be used to assess speech delay. Instead, valuations relied on physicians’ clinical notes regarding the patients’ words, sentences, and overall speech. According to the Denver II Developmental Screening Test, the absence of nonspecific ‘mama’ and ‘dada’ at 12 months; the absence of specific ‘mama’ and ‘dada’ as well as at least two additional words at 18 months; the absence of specific ‘mama’ and ‘dada’ and at least four additional words at 24 months; the lack of two-word combinations and partially intelligible speech at 30 months; and the absence of sentences and at least 10 words at 36 months were considered indicative of abnormal language development [16,17,18]. Speech-related follow-up notes were evaluated according to these developmental cut-off points. Based on this assessment, patients were classified as having either normal or abnormal language development. Children younger than 12 months were excluded from this evaluation. To minimize potential misclassification bias and secure data entry fidelity, a two-stage review process was implemented: all developmental language data were independently extracted from the medical records by one investigator and subsequently verified and cross-confirmed by a second, senior clinical investigator.

The data of the patients included in this study were extracted retrospectively from medical records. No additional diagnostic procedures or interventions were performed; therefore, informed consent was not required.

Statistical analyses were performed using IBM SPSS Statistics for Windows, Version 30.0 (IBM Corp., Armonk, NY, USA). The distribution of continuous variables was assessed using the Kolmogorov–Smirnov test, which demonstrated non-normal distribution. Accordingly, continuous variables are presented as median (minimum–maximum) and interquartile range (25th–75th percentiles), while categorical variables are expressed as frequencies and percentages.

Comparisons of continuous variables between more than two independent groups were performed using the Kruskal–Wallis test. When the Kruskal–Wallis test was statistically significant, post hoc pairwise comparisons were conducted using the Mann–Whitney U test with Bonferroni correction to account for multiple testing. Associations between categorical variables were evaluated using the chi-square test or Fisher’s exact test, as appropriate.

All statistical tests were two-tailed, and a *p*-value < 0.05 was considered statistically significant. For post hoc analyses, the significance threshold was adjusted according to the Bonferroni method. Analyses were performed using all available data for each variable.

## 3. Results

The demographic characteristics of the boys with DMD are summarized in Table 1. The chronological age at the first evaluation demonstrated a non-normal distribution, with a median age of 1.7 years (range: 0.10–3.4; interquartile range [IQR] 25–75: 1.10–2.09). The median age at diagnosis was 15 months (range: 0–36; interquartile range [IQR] 25–75: 9–20). The definitive diagnostic confirmation of the patients was established via genetic testing (MLPA or sequencing) in 99.0% of the cases (*n* = 196), or through a muscle biopsy demonstrating a complete absence of dystrophin expression in the remaining 1.0% (*n* = 2) of patients who presented with negative genetic panels.

The medical investigation was initially triggered by incidental hyperCKemia, which led to diagnosis in 181 patients (91.4%). In comparison, 7 patients (3.5%) were diagnosed following the detection of elevated liver function tests, 9 (4.5%) due to clinical symptoms (such as frequent falls), and one child (0.5%) owing to a known family history of a brother with DMD. CK levels at the time of diagnosis were 10,300 IU/L (range: 1070–39,124; interquartile range [IQR] 25–75: 8151–15,987). Calf muscle pseudohypertrophy was present in 132 boys (66.7%) at the time of diagnosis. Maternal carrier status was confirmed in 52 cases (26.3%). Genetic tracking was successfully completed for all 198 mothers in the cohort; 52 (26.3%) were confirmed as carriers, while 146 (73.7%) tested negative for the familial mutation. A positive family history of DMD was present in 32 cases (16.2%). Although this family history existed beforehand, with the exception of one individual who was directly investigated for DMD due to having an affected brother, the boys were not evaluated at birth due to their asymptomatic status; their diagnostic pathways were initiated later through subsequent incidental hyperCKemia or targeted clinician-led CK screening.

MLPA analysis was performed in all 198 (100%) patients, while comprehensive DMD gene analysis was conducted in 35 (17.8%) boys. WES was performed in 4 (2%) patients during the diagnostic process. According to the genetic results, deletions were present in 154 patients (77.8%), duplications in 15 (7.6%), and point mutations in 27 (13.6%) (Table 2). In two patients, no pathogenic variants consistent with DMD were identified by MLPA, comprehensive DMD gene analysis, or whole-gene sequencing. Of the deletions, 35 (22.7%) were single-exon deletions, 103 (66.9%) were multi-exon deletions, and 16 (10.4%) were large deletions. Among the 154 patients with deletions, 31 (20.1%) were in the proximal hot-spot region, and 109 (70.8%) were in the distal hot-spot region. Deletions outside the hot-spot regions accounted for 4 (2.6%) in the non-hot-spot region and 10 (6.5%) in the intermediate region. Twelve patients (7.8%) had mutations localized in both the proximal hot-spot region and the intermediate region. All these mutations were large deletions, and they were included in the proximal hot-spot region category. Regarding point mutations, nonsense mutations were the most frequent, found in 13 patients (48.1%), followed by frameshift mutations in 4 patients (14.8%) and splice-site mutations in 2 patients (7.4%).

Independent ambulation was documented in the medical records of 160 children. Of these boys, independent walking began at a median age of 16 months (range, 11–34 months; interquartile range [IQR] 25–75: 14–18), and 29 (14.6%) boys started walking at 19 months of age or later. When the age at independent ambulation was compared across deletion size categories (single-exon, multi-exon, and large deletions), no significant difference was observed (Kruskal–Wallis H = 0.788, df = 2, *p* = 0.674). Conversely, the age at independent ambulation differed significantly across hot-spot regions (Kruskal–Wallis H = 8.378, df = 3, *p* = 0.039), indicating an association between mutation localization and walking age. However, post hoc pairwise comparisons using Mann–Whitney U tests with Bonferroni correction revealed no statistically significant differences in age at independent ambulation between specific hot-spot groups (all adjusted *p*-values > 0.008). This lack of localized pairwise significance indicates that the global association must be interpreted with extreme caution, likely reflecting reduced statistical power due to the small sample sizes in the non-hot-spot (*n* = 4) and intermediate (*n* = 10) cohorts rather than a definitive localized phenotype driver.

Language development data were available for 189 children in the clinical files. Language development was delayed for their age in 59 patients (29.8%). Language development did not differ significantly according to deletion size categories (Kruskal–Wallis H  = 2.053, df = 2, *p* = 0.358). Language development did not differ significantly according to DMD gene hot-spot localization (Kruskal–Wallis H = 0.803, df = 3, *p* = 0.849). During follow-up, a total of three patients (1.5%) were diagnosed with autism spectrum disorder (ASD). These ASD diagnoses were extracted retrospectively from the medical records and had been formally established by child and adolescent psychiatrists following professional clinical referral. One of these patients had a nonsense point mutation, whereas the other two had deletions located in the distal (central) hot-spot region.

## 4. Discussion

Early detection of DMD is paramount to initiating early interventions and pathogenetic treatments before significant muscle tissue loss occurs. However, expanding clinical data for this specific under-three age group has historically been challenging. As recently highlighted by Fontanelli et al. in a comprehensive 20-year analysis of neuromuscular clinical research, age representation remains deeply uneven across clinical trials and observational cohorts, often leading to a significant underrepresentation of infants and toddlers [19]. Our study directly addresses this age-specific evidence gap by characterizing the largest single-center cohort of children diagnosed before three years of age in our region, specifically in a country that lacks a universal DMD newborn-screening program. Our study showed that diagnoses before age three were mostly based on elevated CK levels, which is consistent with the literature [20]. Additionally, in early childhood, the presence of pseudohypertrophy at diagnosis in more than half of the children, abnormal early language development in about one third, and delayed achievement of independent walking compared to the general population are significant findings of our study.

Family-based comparative data demonstrate that early diagnosis in DMD is not merely a theoretical advantage but confers clear benefits across multiple domains, including educational and social preparedness, access to supportive services, informed evaluation of therapeutic options, and participation in clinical research [21]. Studies comparing siblings within the same family who received early versus late diagnoses have shown better health and functional outcomes in those diagnosed earlier. Early diagnosis not only influences the motor course of the disease but also provides a critical opportunity for timely neurodevelopmental monitoring, family counseling, and access to treatment [10]. By examining the clinical features of children with DMD diagnosed before age three, our study demonstrates how this early diagnostic window impacts clinical practice and provides clear evidence of the real-world benefits of early diagnosis.

In a study conducted in Austria and Germany, the first symptoms appeared at an average age of 3 years, with difficulty climbing stairs being the most common initial sign [22]. The study noted that the majority of children, particularly during infancy and the pre-walking or early walking stages, were diagnosed incidentally after elevated CK levels were detected. Consistent with these findings, the majority of patients in our cohort were also diagnosed incidentally due to elevated CK levels, and their CK values were comparable to those reported in the literature [9,23]. In addition, the presence of pseudohypertrophy—an indicator of muscle breakdown, similar to elevated CK—in about two-thirds of children diagnosed before age three is another significant finding of our study. The comparison of clinical and genetic characteristics between the present cohort and the key published literature on early-diagnosed DMD populations is presented in Table 3.

The genetic analysis results confirm that deletions are the most common mutation type in DMD. In our cohort, the deletion rate was 77.8%, which is consistent with large series reported in the literature [25,26]. The predominance of multi-exon deletions and their clustering within the distal hotspot region (exons 45–53) mirrors the well-known mutation pattern of the DMD gene [25]. Among point mutations, nonsense mutations were the most common, which is consistent with previous reports [26]. These mutations are known to generate premature stop codons, leading to early truncation of dystrophin and are therefore often associated with a more severe phenotype [27]. Overall, the mutation distribution observed in our study supports international genetic data on the DMD mutation spectrum.

Our study found that independent walking in children with DMD occurs later, and 14.6% of the patients achieved independent ambulation after 18 months of age. Consistent with the literature, this finding demonstrates that delayed attainment of independent ambulation represents one of the strongest early motor discriminative features of DMD [4]. Additionally, these findings demonstrate that DMD has neurodevelopmental effects independent of early muscle damage [5,6,28,29,30]. A third of the patients had delayed language development for their age. The literature explicitly reports that the non-motor components of DMD, including cognitive, behavioral, language, and psychosocial areas, are often overlooked and not adequately addressed early in life [6]. All of these findings suggest that developmental delays could serve as early indicators of DMD and highlight the importance of developmental screenings in infants and early childhood [5,29]. Any male child with developmental delays at any developmental milestone should be considered for DMD [31]. Screening CK measurement is of critical importance in these patients [5].

Regarding the statistical layout, the age at independent ambulation showed a globally significant difference across hot-spot regions (Kruskal–Wallis H = 8.378, df = 3, *p* = 0.039), indicating an association between mutation localization and walking age. Post-hoc pairwise comparisons using Mann–Whitney U tests with Bonferroni correction revealed no statistically significant differences in age at independent ambulation between the individual hot-spot groups (all adjusted *p*-values > 0.008). Therefore, this statistical finding must be interpreted with strict caution. While the global test suggests a potential relationship between mutation localization and early motor milestones, the conservative nature of the Bonferroni correction combined with the low sample density in non-hot-spot cohorts prevents us from concluding that specific deletion domains uniquely dictate early walking delay. Further prospective, large-scale studies with evenly distributed genetic sub-cohorts are required to clarify whether specific hot-spot borders modulate the pre-clinical motor phenotype during infancy.

The international meeting report by Armstrong et al., which focused on the care of children with DMD aged 0–3 years, emphasized that there are still no evidence-based, standardized clinical follow-up protocols or treatment algorithms for this age group [8]. This gap leads to considerable variability among clinicians regarding when treatment is initiated and how patients should be monitored. The report also highlighted that children with DMD at this early age range exhibit developmental delays across nearly all domains, and that conventional motor assessment tools are insufficient for this population [8]. Consequently age-appropriate, early-childhood-specific outcome measures are urgently needed. Our study provides valuable data on the under-characterized period before age three. It makes a significant contribution to the literature by delineating not only motor manifestations but also non-motor features in children diagnosed in early childhood, as similarly underscored in the meeting report. Unfortunately, as in many countries worldwide, the absence of a standardized follow-up algorithm for children diagnosed early in Türkiye remains a critical unmet need. The article titled “Development of the Accredited Duchenne Centers (ADC) Program,” published by Groot et al. in 2025, demonstrates that early diagnosis in DMD gains true clinical significance only when accompanied by standardized care delivered in multidisciplinary, guideline-adherent centers, and that referral of patients diagnosed at an early age to such centers is critical for long-term outcomes [32].

The cohort study by Brogna et al. demonstrates that central nervous system involvement in DMD is common, highlighting the inadequacy of considering the disease solely as a motor disorder [24]. The identification of at least one neurodevelopmental or psychiatric disorder in more than one-third of patients points to central nervous system-related manifestations that begin early but are frequently recognized later in life. The weak genotype–phenotype correlation observed in the cohort suggests that these findings may occur independently of mutation type, supporting the notion that all children with DMD should be considered candidates for early neurodevelopmental assessment [27]. Consistent with these observations, our study found delayed language development in approximately one-third of the children and identified three patients who were being followed with a diagnosis of ASD, underscoring the importance of close developmental surveillance in boys with DMD during early childhood and the potential benefits of early intervention in supporting later developmental outcomes. Because our study cohort was exceptionally young—with all children evaluated under three of age—other neurodevelopmental and behavioral disorders that typically manifest later in childhood, such as attention-deficit/hyperactivity disorder (ADHD) or mild intellectual disability, could not be formally diagnosed or assessed during this early age window. The long-term monitoring of these emergent behavioral phenotypes remains a distinct focus of our ongoing clinical research and will be addressed as a separate study topic in the future.

This study has several limitations. Because of its retrospective design, language development was assessed from physicians’ notes using the Denver II criteria, which precluded the use of standardized, objective neurodevelopmental tests. It is certain that this retrospective adaptation of the Denver II milestones using clinical records cannot fully replace a comprehensive, face-to-face standardized diagnostic test battery administered prospectively by a dedicated development specialist. This lack of direct standardized laboratory testing represents an inherent limitation in interpreting the absolute depth of the expressive and receptive communication delays within our population, highlighting a key area that should be explored via standardized prospective cognitive testing in future clinical investigations. The relatively short follow-up period also restricted the assessment of long-term motor and functional outcomes.

Furthermore, our findings should be evaluated with consideration of certain demographic boundaries. Data regarding race and ethnicity could not be incorporated due to regional medical recording regulations that prohibit the tracking of these protected categories in routine hospital registries. While our cohort represents a homogeneous regional population of Turkish infants, future global multi-center trials are required to determine whether these early diagnostic triggers and clinical phenotypes vary across diverse ethnic and racial sub-groups. Additionally, due to the retrospective nature of the chart review, the precise longitudinal timing and exact chronological dates of the initial serum CK measurements were not uniformly documented across the entire cohort, preventing a detailed sub-analysis of CK fluctuations stratified strictly by minor age groups or specific months of life. However, a significant strength of this study is the thorough presentation of genetic and clinical data from a large cohort of patients diagnosed at an early age. Furthermore, given that the cohort was evaluated at a very young age (under three years), establishing definitive genotype–phenotype correlations regarding early laboratory findings and clinical phenotypes was not statistically robust at this stage. Longitudinal, long-term follow-up of these children is currently underway, and comprehensive genotype–phenotype analyses will be addressed in future prospective publications as the cohort transitions into later childhood.

While the clinical patterns identified in our cohort do not represent a fundamental scientific breakthrough but rather confirm established global trends within a specific, regional population, the true value of this study lies in its practical and epidemiological significance within our healthcare setting. To date, this work constitutes the first and largest single-center cohort focusing exclusively on DMD patients under 36 months of age in Türkiye, bridging a major regional data gap. Documenting that 91.4% of these infants were diagnosed through accidental hyperCKemia highlights a critical local reality: in the absence of a universal newborn screening program, early detection relies almost entirely on incidental laboratory workflows. Providing these regional statistics offers a necessary, real-world baseline for local healthcare policymakers working toward optimizing infant developmental surveillance and standardizing diagnostic routing pathways in our region.

From a future clinical perspective, these insights underscore the critical need to establish concrete clinical action pathways in pediatric primary care. First, primary care pediatricians and family physicians should abandon traditional ‘wait-and-see’ strategies for early milestones lag; instead, a screening serum CK panel should be routinely ordered for any young male child presenting with unexplained speech delays or global developmental delays. Second, because early identification only achieves its true translational potential when paired with immediate, specialized intervention, multidisciplinary neuromuscular centers must standardize the integration of formal, age-appropriate neurodevelopmental screening tools (such as the Denver II or Bayley Scales) into their routine follow-up protocols from the very moment of diagnosis. Finally, while optimizing clinical awareness helps reduce the diagnostic gap, the establishment of universal national newborn screening (NBS) programs for DMD remains the ultimate definitive strategy. Universal NBS guarantees pre-symptomatic detection, facilitates proactive neuroprotective and genetic family counseling, and secures immediate eligibility for emerging disease-modifying and gene-targeted therapies before irreversible mechanical muscle and neural tissue loss occurs.

## 5. Conclusions

These findings emphasize that earlier diagnosis of DMD is possible and that the age at diagnosis is decreasing due to increased global awareness. Our findings suggest that these children may exhibit significant impairments in early language development and broader neurodevelopmental processes. Early diagnosis and interventions can positively influence the disease progression and improve long-term outcomes. These findings indicate that CK measurement should be more widely considered in childhood, particularly in children presenting with developmental delay. Furthermore, neurodevelopmental follow-up should be standardized as an integral component of DMD management after diagnosis.

## Figures and Tables

**Table 1:** Demographic characteristics of boys with Duchenne Muscular Dystrophy (DMD) (*n* = 198).

Demographic Characteristics	Value / *n* (%)
Age at first evaluation (years, median (min-max))	1.7 (0.1–3.4)
Age at diagnosis (months, median (min-max))	15 (0–36)
Methods of diagnosis (*n* (%))	
CK elevation	181 (91.4)
Transaminase elevation	7 (3.5)
Clinical symptoms	9 (4.5)
DMD in the brother	1 (0.5)
CK levels at the time of diagnosis (U/L, median (min-max))	10,300 (1070–39,124)
Calf muscle pseudohypertrophy at first evaluation (*n* (%))	132 (66.7)
Carrier mother (*n* (%))	52 (26.3)
Family history of DMD (*n* (%))	32 (16.2)
Independent walking age (months, median (min-max)) (*n* = 160)	16 (11–34)
Language developmental delay (*n* (%)) (*n* = 189)	59 (29.8)

CK: Creatine Kinase, DMD: Duchenne Muscular Dystrophy.

**Table 2:** Genetic characteristics of boys with Duchenne Muscular Dystrophy (DMD).

Genetic Features	*n* (%)
Genetic results (*n* = 198)	
Deletion	154 (77.8)
Duplication	15 (7.6)
Point mutation	27 (13.6)
No mutation was detected	2 (1)
Structural classification of the deletions (*n* = 154)	
Single-exon deletions	35 (22.7)
Multi-exon deletions	103 (66.9)
Large deletions	16 (10.4)
Genomic localization of the deletions (*n* = 154)	
Proximal hot-spot region	31 (20.1)
Proximal hot-spot and intermediate regions	12 (7.8)
Distal hot-spot regions	109 (70.8)
Non-hot-spot regions	4 (2.6)
Intermediate regions	10 (6.5)
Type of point mutations (*n* = 27)	
Nonsense mutation	13 (48.1)
Frameshift mutation	4 (14.8)
Splice-site mutation	2 (7.4)

**Table 3:** Comparison of clinical and genetic characteristics between the present cohort and the key published literature on early-diagnosed DMD populations.

Study Feature	Present Cohort (*n* = 198)	Hiebeler et al. (2023) [22]	Hoskens et al. (2024) [5]	Brogna et al. (2025) [24]
Geographic Region	Türkiye	Austria & Germany	Systematic Review	Italy
Target Age Group	<3 years	All pediatric (Time to dx)	Infancy & Early Childhood	Pediatric to Adult
Primary Diagnostic Trigger	Incidental CK elevation (91.4%)	Incidental CK in infants/toddlers	Gross Motor/ Global delay	Neurodevelopmental/ Psychiatric signs
Median Age at Diagnosis	15 months	3 years (first clinical signs)	Variable across registries	>3 years (for neurobehavioral dx)
Language Delay Rate	29.8%	Not explicitly tracked	Prominent in infant models	33.3 % (Central nervous cohort)
Key Clinical Focus	Early CK screening for delays	Reducing time to diagnosis	Standardized testing (0–3 years)	Early neurodevelopmental surveillance

## Data Availability

Anonymized patient-level data are available upon reasonable request from the corresponding author at anurcoskun@gmail.com.

## References

[1] Duan D., Goemans N., Takeda S., Mercuri E., Aartsma-Rus A. (2021). Duchenne muscular dystrophy. Nat. Rev. Dis. Prim..

[2] Fortunato F., Farnè M., Ferlini A. (2021). The DMD gene and therapeutic approaches to restore dystrophin. Neuromuscul. Disord..

[3] Tyler K.L. (2003). Origins and early descriptions of “Duchenne muscular dystrophy”. Muscle Nerve.

[4] Norcia G., Lucibello S., Coratti G., Onesimo R., Pede E., Ferrantini G., Brogna C., Cicala G., Carnicella S., Forcina N. (2021). Early Gross Motor Milestones in Duchenne Muscular Dystrophy. J. Neuromuscul. Dis..

[5] Hoskens J., Paulussen S., Goemans N., Feys H., De Waele L., Klingels K. (2024). Early motor, cognitive, language, behavioural and social emotional development in infants and young boys with Duchenne Muscular Dystrophy—A systematic review. Eur. J. Paediatr. Neurol..

[6] Lorentzos M., Parsons J.A., Jones K.J., Servais L. (2024). Early diagnosis of Duchenne muscular dystrophy—A Treat-NMD international workshop. Neuromuscul. Disord..

[7] Aartsma-Rus A., Hegde M., Ben-Omran T., Buccella F., Ferlini A., Gallano P., Howell R.R., Leturcq F., Martin A.S., Potulska-Chromik A. (2019). Evidence-Based Consensus and Systematic Review on Reducing the Time to Diagnosis of Duchenne Muscular Dystrophy. J. Pediatr..

[8] Armstrong N., Apkon S., Berggren K.N., Braun C., Ciafaloni E., Connolly A., Kennedy A., Kuntz N., Mathews K., McGuire M. (2024). The Early Care (0–3 Years) In Duchenne Muscular Dystrophy Meeting Report. J. Neuromuscul. Dis..

[9] Rohlenová M., Machová K., Baranová J., Mokrá L., Mensová L., Mazanec R., Juříková L., Staněk J., Fuchsová P., Lauerová B. (2025). Serum Creatine Kinase and Transaminase Levels in Duchenne and Becker Muscular Dystrophies. Muscle Nerve.

[10] Mercuri E., Pane M., Cicala G., Brogna C., Ciafaloni E. (2023). Detecting early signs in Duchenne muscular dystrophy: Comprehensive review and diagnostic implications. Front. Pediatr..

[11] Muntoni F., Torelli S., Ferlini A. (2003). Dystrophin and mutations: One gene, several proteins, multiple phenotypes. Lancet Neurol..

[12] Carls C., Krajacic P. (2017). Bridging the Gap: An Osteopathic Primary Care-Centered Approach to Duchenne Muscular Dystrophy. J. Am. Osteopath. Assoc..

[13] Gloss D., Moxley R.T., Ashwal S., Oskoui M. (2016). Practice guideline update summary: Corticosteroid treatment of Duchenne muscular dystrophy: Report of the Guideline Development Subcommittee of the American Academy of Neurology. Neurology.

[14] Bladen C.L., Salgado D., Monges S., Foncuberta M.E., Kekou K., Kosma K., Dawkins H., Lamont L., Roy A.J., Chamova T. (2015). The TREAT-NMD DMD Global Database: Analysis of more than 7000 Duchenne muscular dystrophy mutations. Hum. Mutat..

[15] Li Y., Liu Z., OuYang S., Zhu Y., Wang L., Wu J. (2016). Distribution of dystrophin gene deletions in a Chinese population. J. Int. Med. Res..

[16] Anlar B., Bayoğlu B., Yalaz K. (2009). Denver II Gelişimsel Tarama Testi “Türk Çocuklarina Uyarlanmasi ve Standardizasyonu”.

[17] Frankenburg W.K., Dodds J.B. (1967). The Denver developmental screening test. J. Pediatr..

[18] Borowitz K.C., Glascoe F.P. (1986). Sensitivity of the Denver Developmental Screening Test in speech and language screening. Pediatrics.

[19] Fontanelli L., Vadi G., Bellini G., Cossu A., Siciliano G. (2025). Equity in neuromuscular research: A 20-year analysis of race, ethnicity, sex, and age representation. J. Neurol..

[20] Zygmunt A.M., Wong B.L., Horn P.S., Lambert J., Bange J.E., Rybalsky I., Chouteau W., Tian C. (2023). A longitudinal study of creatine kinase and creatinine levels in Duchenne muscular dystrophy. Muscle Nerve.

[21] Bhattacharyya O., Campoamor N.B., Armstrong N., Freed M., Schrader R., Crossnohere N.L., Bridges J.F.P. (2024). Assessing the Benefits and Harms Associated with Early Diagnosis from the Perspective of Parents with Multiple Children Diagnosed with Duchenne Muscular Dystrophy. Int. J. Neonatal Screen..

[22] Hiebeler M., Thiele S., Reilich P., Bernert G., Walter M.C. (2023). Time to diagnosis of Duchenne muscular dystrophy in Austria and Germany. Sci. Rep..

[23] Dangouloff T., Lang H., Benmhammed N., Servais L. (2025). Newborn screening and rapid genomic diagnosis of neuromuscular diseases. J. Neuromuscul. Dis..

[24] Brogna C., Moriconi F., Capasso A., Arpaia C., Cicala G., Ricci M., Villa M., Pellizzari M., De Gioia A., Turano M. (2025). Identification and treatment of neurodevelopmental and mental disorders in boys and adults with Duchenne muscular dystrophy: A cohort study. Arch. Dis. Child..

[25] Ge L., Yang Y., Yang Y., Chen Y., Tao N., Zhang L., Zhao C., Zhang X. (2024). DMD mutations in pediatric patients with phenotypes of Duchenne/Becker muscular dystrophy. Open Med..

[26] Sparber P.A., Zinina E.V., Shchagina O., Polyakov A.V., Kutsev S.I. (2025). Molecular and genetic characteristics of patients from the National Registry of Duchenne/Becker Muscular Dystrophy in the Russian Federation: Pilot analysis. J. Neuromuscul. Dis..

[27] Soares F.M., Rosa B.F., Giordani G.M., Rocha D.L., Brusius-Facchin A.C., Becker M.M., Saute J.A.M. (2025). Genotype-phenotype correlation of neurodevelopmental disorders in patients with dystrophinopathies. J. Pediatr..

[28] Connolly A.M., Florence J.M., Cradock M.M., Malkus E.C., Schierbecker J.R., Siener C.A., Wulf C.O., Anand P., Golumbek P.T., Zaidman C.M. (2013). Motor and cognitive assessment of infants and young boys with Duchenne Muscular Dystrophy: Results from the Muscular Dystrophy Association DMD Clinical Research Network. Neuromuscul. Disord..

[29] van Dommelen P., van Dijk O., de Wilde J.A., Verkerk P.H. (2024). Short developmental milestone risk assessment tool to identify Duchenne muscular dystrophy in primary care. Orphanet J. Rare Dis..

[30] van Dommelen P., van Dijk O., de Wilde J.A., Verkerk P.H. (2020). Early developmental milestones in Duchenne muscular dystrophy. Dev. Med. Child. Neurol..

[31] Armstrong N., Schrader R., Fischer R., Crossnohere N. (2022). Duchenne expert physician perspectives on Duchenne newborn screening and early Duchenne care. Am. J. Med. Genet. C Semin. Med. Genet..

[32] de Groot I.J.M., Podolská K., Goemans N., Bakker S.A., Lalout N., Jansen M., Vroom E. (2025). Development of the accredited duchenne centers program, a global program to achieve uniform and up-to-date care for all people living with duchenne muscular dystrophy. Orphanet J. Rare Dis..

